# Acrocyanosis and digital necrosis are associated with poor prognosis in COVID‐19

**DOI:** 10.1002/ccr3.3276

**Published:** 2020-08-30

**Authors:** Guitti Pourdowlat, Zohre Naderi, Farhad Seif, Davood Mansouri, Hanieh Raji

**Affiliations:** ^1^ Chronic Respiratory Diseases Research Center National Research Institute of Tuberculosis and Lung Diseases Shahid Beheshti University of Medical Sciences Tehran Iran; ^2^ Department of Pulmonology Isfahan University of Medical Sciences Isfahan Iran; ^3^ Department of Immunology and Allergy Academic Center for Education, Culture, and Research Tehran Iran; ^4^ Neuroscience Research Center Iran University of Medical Sciences Tehran Iran; ^5^ Department of Clinical Immunology and Infectious Diseases National Research Institute of Tuberculosis and Lung Diseases Shahid Beheshti University of Medical Sciences Tehran Iran; ^6^ The Clinical Tuberculosis and Epidemiology Research Center National Research Institute of Tuberculosis and Lung Diseases Shahid Beheshti University of Medical Sciences Tehran Iran; ^7^ Pediatric Respiratory Diseases Research Center National Research Institute of Tuberculosis and Lung Diseases Shahid Beheshti University of Medical Sciences Tehran Iran; ^8^ Department of Internal Medicine Air pollution and Respiratory Diseases Research Center Ahvaz Jundishapur University of Medical Sciences Ahvaz Iran

**Keywords:** acrocyanosis, COVID‐19, digital necrosis, interleukin‐6, noradrenaline

## Abstract

Acrocyanosis and digital necrosis, which caused by microangiopathic and immunothrombosis phenomenon, may accompanied by microvascular involvement of other organs. Therefore, this finding can play a prognostic role in covid‐19 outcome.

## Graphical abstract

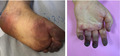

## 1 INTRODUCTION

Coronavirus infection disease 2019 (COVID‐19) may present with different symptoms and complications. Acrocyanosis and digital necrosis may be associated with COVID‐19. We describe two patients with COVID‐19 who died with acrocyanosis and digital necrosis at the terminal stage of their illness.

In the last days of 2019, severe acute respiratory syndrome coronavirus‐2 (SARS‐CoV‐2) caused a disease in Wuhan, China, which was later named as coronavirus infection disease 2019 (COVID‐19) and became a global health problem.^1^ COVID‐19 can present with mild flu‐like to severe symptoms such as acute respiratory distress syndrome (ARDS), septic shock, poorly controlled metabolic acidosis, coagulation dysfunction, and multi‐organ failure.^2^ Therefore, understanding specific and different symptoms and signs of the disease is of great importance. To the best of our knowledge, many of the symptoms and complications corresponded to the COVID‐19 are caused by cytokines and mediators of the immune system induced by the infection. In this regard, cytokine storm is the result of the overproduction of major proinflammatory cytokines such as TNF‐α, IL‐6, and IL‐1β that can produce hypercoagulable state and multiple organ dysfunction.^3^

Similar to other types of ARDS, there is a hypercoagulable state in COVID‐19 patients. This inflammatory condition, as a result of endovascular damage, increased platelet activity, and coagulation cascade causes the phenomenon of immunothrombosis.^4^ Consequently, clot formation can be observed in large and small blood vessels, along with in situ pulmonary thrombosis and also thromboembolism (PTE).^5^ In other words, overactivation of the coagulation pathway that occurs during the time of cytokine storm may be the result of increased thrombin activities. Furthermore, thrombin can play other roles in the inflammatory process by proteinase‐activated receptors (PARs).^3^ On the other hand, Yan Zhang et al reported antiphospholipid syndrome (APS) in COVID‐19.^6^ Livedo reticularis, that also has been reported in COVID‐19,^7^ is related to APS and cold agglutinin disease; accordingly, viral infection is one of the causes of cold agglutinin formation.^8^ Therefore, cold hemagglutinin disease should also be considered in patients with COVID‐19 who present with this skin manifestation.

Furthermore, acrocyanosis has been described in critically ill patients with COVID‐19 because of excessive coagulation status.^9^ Indeed, acrocyanosis and digital necrosis have previously been reported in many rheumatologic disorders and vasculitis, including ANCA‐associated vasculitis, cryoglobulinemia vasculitis, lupus‐related vasculitis, vasculopathy caused by APS, and also fingertips necrosis due to scleroderma vascular involvement as well as Raynaud's phenomenon.^10^, ^11^, ^12^ From a physiologic point of view, gangrene may occur by impaired blood flow and insufficient healing process of digital wounds which is associated with the increasing levels of C‐reactive protein (CRP).^11^ Here, we describe two cases of critically ill patients with COVID‐19 who developed digital acrocyanosis and subsequently fingertips necrosis during their illness.

## 2 CASE 1

**Figure 1:**
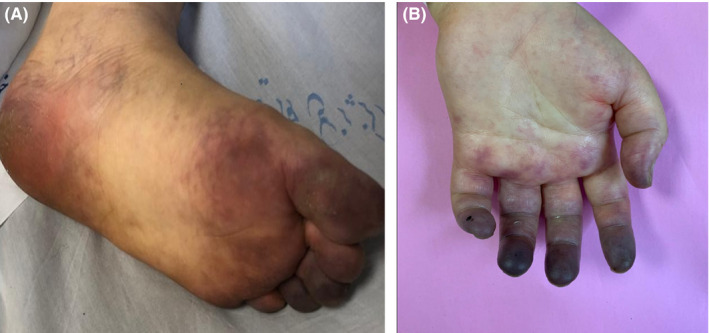
Acrocyanosis and gangrene of the toes (A) and fingers (B) are shown

A 56‐year‐old woman with diabetes referred to the emergency department, with complaints of cough, headache, high‐grade fever, dyspnea, and hypoxemia on April 2020. Chest computed tomography (CT) showed bilateral ground‐glass opacities. Laboratory results revealed normal complete blood count, high CRP titer, and LDH (904 IU/L), while PT, INR, and PTT were in the normal range. Nasopharyngeal sampling was positive for SARS‐CoV‐2 using real‐time PCR method. Supplemental oxygen, prophylactic unfractionated heparin, and hydroxychloroquine were initiated. On day 5 of admission, she was intubated due to respiratory distress and sedative drugs were used to control the condition. On day 6, ecchymosis and patchy skin lesions appeared on her extremities. Owing to the probability of bacterial superinfection, blood and tracheal secretion cultures were performed and meropenem and vancomycin were added to hydroxychloroquine. On day 7, she was transferred to designated COVID‐19 center. At the admission, she was unconscious and still intubated under mechanical ventilation using SIMV + PS mode. The blood pressure and heart rate were 90/65 mm Hg and 87/min, respectively. Atrial blood gas (ABG) evaluation revealed that pO2 was 61 mm Hg and oxygen saturation was 90% with 50% FIO2. Meanwhile, cyanosis was seen at the end of her upper and lower extremities, toes (Figure 1A), and fingers (Figure 1B), and spO2 with digital pulse oximeter was about 87%. Blood sampling showed leukocytosis (16 200 per mm^3^), lymphopenia (1200 per mm^3^), thrombocytopenia (91 000 per mm^3^), Hb (7.8 g/L), high CRP titer (107 mg/L), LDH (1117 IU/L), D‐dimer (7780 µg/mL), ferritin (1650 ng/mL), and IL‐6 (359 pg/mL). Additional laboratory findings indicated negative PANCA, CANCA, anticardiolipin, lupus anticoagulant, and β_2‐_microglobulin. Echocardiographic was normal, while electrocardiogram showed inverted T in V1 to V5. Atorvastatin and aspirin were added to the treatment regimen, and the dose of heparin was increased to the therapeutic dose. For improvement of blood flow on extremities, nitroglycerin ointment was also applied. In spite of the multiple treatment modalities, her clinical condition deteriorated and she ultimately demised.

## 3 CASE 2

A 67‐year‐old woman with a history of type II diabetes mellitus and systemic hypertension developed respiratory distress and was evaluated in the emergency department. Respiratory rate was 36/min, temperature 37.5°C and oxygen saturation 65% on room air. Upon physical examination, coarse end‐inspiratory crackles were noted at both lung bases. Chest X‐ray showed bilateral airspace opacities, and diffuse ground‐glass opacity was also found in spiral chest CT scan. Laboratory findings revealed leukopenia (4200 per mm^3^), lymphopenia (800 per mm^3^), normal platelet (160,000 per mm^3^) and hemoglobin level (13.7 g/dL), high CRP (70 mg/L), LDH (840 IU/L), and D‐dimer (976 µg/mL), whereas PT, INR, and PTT were in the normal ranges. Nasopharyngeal sampling was positive for SARS‐CoV‐2 using real‐time PCR method. She required endotracheal intubation because of no response to oxygen therapy. Sedatives and neuromuscular blockade were used to enforce a lung‐protective low‐stretch strategy. Her treatment was started with chloroquine 300 mg twice daily on the first day, followed by Lopinavir/ritonavir 400/100 mg twice daily. Other prescribed drugs included methylprednisolone, prophylactic unfractionated heparin, and antibiotics. On 9th day of mechanical ventilation, in spite of the improvement in oxygenation and decrease of positive end‐expiratory pressure (PEEP) pressure, the patient developed terminal cyanosis in all fingers of her hands, and a lack of pulse in both radial arteries. Blood pressure decreased, and after initiating the therapeutic dose of heparin and fluid therapy, she had a radial artery pulse in the left hand without any improvement in acrocyanosis. One day later, she developed subcutaneous emphysema in her chest, breasts, and face, and finally, passed away.

## 4 DISCUSSION

Acro‐ischemia and cyanosis have been explained in critically ill patients with COVID‐19 due to excessive coagulation status.^9^ In addition, digital ischemia and necrosis may be observed in other conditions such as diabetes mellitus or drug complications. In this regard, some critically ill patients may need norepinephrine for the treatment of hemodynamic impairment in the ICU. In this condition, high‐dose norepinephrine may be needed for circulatory shock, which can lead to more complications and poor outcomes. Accordingly, a previous history of peripheral vascular disease, hemodynamic disturbances, and prolonged hypotension that can be associated with concomitant use of other drugs such as dopamine may increase the risk of developing norepinephrine‐induced digital necrosis.^13^ Although it is not clear that up to which dose norepinephrine is tolerable, the risk of impaired blood flow and digital necrosis increases when higher doses are administered because of high vasoconstriction effect.^13^

Based on our findings and literature review, this is a multifactorial problem and various issues need to be considered the causes of this condition. Both of our patients suffered from type II diabetes mellitus, and diabetes mellitus is a predisposing factor for ischemia and necrosis of extremities and digits. According to a report from China, the mortality rate was 7.3% in patients with COVID‐19 who suffers from diabetes compared to the total mortality rate of 2.3% in normal population. It seems that diabetes and other associated comorbidities are aggravating factors for disease severity and complications.^14^ To the best of our knowledge, one of the most important long‐term complications of diabetes is microangiopathy, which causes damage to organs such as the eyes, kidneys, skin, and neurons. On the other hand, overexpression of some cytokines, for example, vascular endothelial growth factor (VEGF) and its receptor (VEGF‐R) and the prosclerotic cytokine of transforming growth factor‐β (TGF‐β) play critical roles in angiogenesis and angiopathic transformation in these patients.^15^

Diabetic vasculopathy may be the reason for an impaired contribution of humoral immunity, and metabolic and hemodynamic conditions.^16^ Further, complement‐mediated thrombotic microangiopathies (TMA) have been reported in patients with COVID‐19 that may be a concomitant problem in these patients.^17^ With this respect, microvascular involvement such as inflammatory accumulation of neutrophils and platelets and intravascular fibrin deposition have been also reported in autopsy studies.^18^ Consequently, the occurrence of these risk factors may worsen the disease and complications may be aggravated. Therefore, it is vital to take them into consideration.

In our patients, based on the ECG changes and hemodynamics findings, it seems that acrocyanosis and digital necrosis may be accompanied by vascular and thrombotic events in other organs but there was not enough time for systemic vascular evaluation by Doppler examination to confirm these hypotheses owing to the critical condition of the patients. We noticed that none of our patients received vasoactive drugs in the course of their disease, and in case of receiving a vasopressor, drug side effects might be an additive factor for digital necrosis. The presence of acrocyanosis in patients with COVID‐19 can indicate organ damage due to vascular involvement and is a sign of the severity of the disease. Acrocyanosis can play a prognostic role and demonstrate poor outcomes in these patients. We recommend changing the prophylactic dose to intermediate or therapeutic dose of anticoagulant drugs, possibly plus adequate vasodilator therapy, if the patient presented with acrocyanosis. According to the recent study, catecholamines may play a role in increasing cytokines such as IL‐6,^19^ which can aggravate the complications of COVID‐19.

In conclusion, some important risk factors that increase the chances of microangiopathy and vascular disorders should be considered in critically ill patients with COVID‐19. These items include diabetes mellitus, smoking, a history of atherosclerosis, and high plasma levels of CPR, IL‐6, D‐dimer, and fibrinogen. Moreover, the administration of noradrenaline may be a risk factor for causing acrocyanosis, digital necrosis, and cytokine release via IL‐6 production.

## CONFLICT OF INTEREST

The authors declare no conflict of interest.

## AUTHOR CONTRIBUTION

Both the authors: made substantial contribution to the preparation of this manuscript and approved the final version for submission. GP: did the literature search, drafted the initial version of the manuscript, and revised the manuscript for critically important intellectual content. ZN: report the case 1 and acquired images. FS and DM: revised the manuscript for critically important intellectual content. HR: reported case2, participated in drafting of cases, and she has corresponded.

## ETHICAL APPROVAL

The written consent of IR.MUI.MED.REC.1399.217 was accepted for publishing of this article.
